# Enhancing Corrosion and Wear Resistance of Aluminum Bronze Alloy by Nanosecond Pulsed Laser Surface Melting

**DOI:** 10.3390/ma19183995

**Published:** 2026-09-19

**Authors:** Lingyu Guo, Haojun Cheng, Fu Li, Qing Teng, Kaixiong Hu, Mingxing Han, Yun Chen

**Affiliations:** School of Transportation and Logistics Engineering, Wuhan University of Technology, Wuhan 430063, China

**Keywords:** aluminum bronze alloy, nanosecond pulsed laser, phase transition, corrosion, wear

## Abstract

Aluminum bronze alloys are widely used in marine engineering components due to their good mechanical properties, wear resistance, and corrosion resistance. However, surface degradation such as wear and corrosion remains a challenge during long-term service. In this study, nanosecond pulsed laser surface melting (LSM) was applied to an aluminum bronze alloy at different laser powers to improve its surface performance. The results show that LSM treatment leads to the formation of a remelted layer with increased content of the martensitic *β*’ phase and reduced *α* phase. The sample treated at 140 W (L2) exhibited the highest surface hardness (186.957 HV, 36.42% higher than that of the untreated sample) and the smallest wear scar width (763.5 μm, a 24.33% reduction). The L2 also achieved the best corrosion resistance, with a corrosion potential of −0.26515 V (increased by 0.03 V), corrosion current density of 5.34145 μA/cm^2^, and polarization resistance of 3382.9 Ω·cm^2^. The improved performance is attributed to the formation of martensite and the increase in aluminum-rich phases on the surface.

## 1. Introduction

Aluminum bronze is a copper alloy with aluminum as the main strengthening element [1]. Due to its excellent mechanical properties, good wear resistance and corrosion resistance, it is widely used in civil and military industries such as oil and gas platform pumps, valves, and gears. Especially in offshore engineering structures, it has a long service history and a wide range of applications, including but not limited to ship propellers, pump blades, seawater pipe fittings, etc. [2,3]. A large number of studies have shown that damages such as friction and wear, electrochemical corrosion and cavitation corrosion often occur on the surface of aluminum bronze during service [4,5]. Therefore, it is crucial to improve the wear resistance and corrosion resistance of the aluminum bronze surface.

In practical applications, the parts in marine engineering structures are often formed by casting. The phase composition of as-cast aluminum bronze alloy is relatively complex, mainly consisting of the *α*-Cu solid solution phase, the residual martensite *β*’ phase, and some *κ* phases [6]. Among them, the *α* phase belongs to the face-centered cubic structure, which has good ductility and low strength; the *β*’ phase is a metastable phase formed when the *β* phase transforms due to a too-fast cooling rate (the *β* phase transforms into *α* + *γ*_2_ eutectoid during slow cooling). This phase increases the strength of the alloy and reduces its plasticity, and it is also one of the main strengthening phases in the current application of aluminum bronze; the main component of the *κ* phase is Fe_3_Al, and the grains are granular or petal-shaped, distributed in the *α* phase and *β*’ phase. In the service environment represented by seawater, the matrix *α* phase is corroded first, and then the *κ* phase with high Al content precipitates, forming a dense protective film containing Al and Cu (mainly Cu_2_O and Al_2_O_3_) on the surface. This protective film gives aluminum bronze good corrosion resistance [7].

To improve the wear resistance and corrosion resistance of the aluminum bronze alloy surface, many scholars have conducted extensive research in the fields of processes such as laser cladding [8], laser peening [9], laser surface quenching [10], friction stir processing [11], and electrodeposition [12]. Among them, laser surface melting (LSM) has been widely used in the surface modification of copper and copper alloys due to its advantages such as non-contact processing, high flexibility, precise control, and a small heat-affected zone. Tang et al. [13] performed LSM treatment on manganese-nickel aluminum bronze using a 2 kW continuous laser with different scanning speeds and spot diameters, producing a remelted layer several hundred micrometers thick on the surface. Under the optimal laser parameters, the cavitation erosion resistance can be increased by 5.8 times. Song et al. [14] carried out LSM treatment on cast nickel-aluminum bronze using a 0.8 kW continuous laser, obtaining a remelted layer of 720 μm with fine equiaxed and dendritic microstructures, and the corrosion rate was reduced by 25%. Zeng et al. [15] treated the surface of nickel-aluminum bronze using LSM technology. Through the synergistic mechanism of grain refinement strengthening, dislocation strengthening, and precipitation strengthening, a uniform martensite structure was obtained, which reduced the selective corrosion on the surface of nickel-aluminum bronze and effectively improved its corrosion resistance. Song et al. [16] performed LSM treatment on cast manganese aluminum bronze using a continuous laser, obtaining a fine single-phase remelted layer with a homogenized microstructure, and the corrosion rate was reduced by 51.35% while the CE rate was about 3.07 times lower than that of the cast sample. Liu et al. [17] prepared a Ni_60_A composite coating on nickel-aluminum bronze by plasma spraying combined with laser cladding, and at an optimal laser power of 2000 W, the coating exhibited a maximum microhardness of 913.13 HV_0.5_ (about 4.5 times that of the substrate) and the best corrosion resistance with a corrosion current density as low as 2.899 × 10^−7^ A·cm^−2^. However, there has been little research on the effect of pulsed laser surface melting on the corrosion and wear resistance of aluminum bronze alloy.

In the present work, the effects of pulsed laser surface melting with different laser powers on the modification of surface morphology, structural transformation, corrosion, and wear resistance of an aluminum bronze alloy were systematically studied. The relationships among the laser power, microstructure, and properties are discussed in depth in this work, and an optimal parameter can be obtained in pulsed laser processing. This study can provide a theoretical basis and technical support for the performance regulation of aluminum bronze alloys.

## 2. Materials and Methods

The materials used in the experiment were aluminum bronze plates supplied by Fengdi Metal Materials Company, Foshan, China. The plates were received as untreated raw material without subsequent heat treatment, corresponding to the as-cast condition. The measured main elemental composition is shown in Table 1, with deviations from the nominal specification as noted. The plates were cut into specimens with dimensions of 15 mm × 15 mm × 2 mm by the wire-cutting process.

The plates were locally treated using the high-power pulsed fiber lasers produced by Shenzhen JPT Opto-electronics Co., Ltd., Shenzhen, China (YDFLP-E2-200-M7-M-R). The specific experimental parameters are as follows: laser powers of 120 W, 140 W, and 160 W; scanning speed of 3000 mm/s; frequency of 1000 kHz; pulse width of 200 ns; wavelength of 1064 nm; line spacing of 0.015 mm; number of repetitions of 5; and spot diameter of 0.08 mm. The laser-treated area is 1.5 cm × 1.5 cm. The beam profile was Gaussian, and the spot overlap ratio was 96.25%. For the convenience of description in the following text, the original specimen is labeled as L0, and the specimens treated by pulsed laser are labeled as L1, L2, and L3 in the order of increasing power. The relevant data on the methods for different samples are presented in Table 2. The pulsed laser energy density calculation formula is as follows:

(1) $$E=\frac{Q}{S}=\frac{4Q}{\pi D^{2}}=\frac{4P}{\pi D^{2}f}$$

where *E* is the laser energy density (J/cm^2^), *Q* is the single pulse energy (J), *S* and *D* represent the laser spot area (cm^2^) and diameter (cm), respectively, *P* is the laser average power (W), and *f* is the laser frequency (Hz).

The surface morphology of the samples was examined by scanning electron microscopy (Thermo Fischer, Waltham, MA, USA, SEM, Quanta FEG 450). The structure of all the samples was examined by X-ray diffraction (Bruker AXS GmbH, Karlsruhe, Germany, XRD, D8 ADVANCE) with Cu Kα radiation (*λ* = 0.154056 nm). The XRD test range was set from 20° to 100°, and the scanning speed was 3 °/min. The XPS analysis was performed by X-ray photoelectron spectroscopy (Thermo Fischer, Waltham, MA, USA, ESCALAB Xi+) using Al-Kα X-ray (1486.6 eV). The working potential was 12.5 kV, and the filament current was 16 mA. The counts were integrated over 10 cycles. The pass energy was 40 eV, and the step was 0.1 eV. The electron emission angle was 45°. The size of the test area was a 500 μm circle, and the etched area was a 1.6 mm circle. During spectra acquisition, the base pressure was 8 × 10^−10^ Pa. A charge neutralizer was used. An etching rate of 1 nm/s was applied to characterize the element depth profile.

The hardness of the experimental specimens was measured using a microhardness tester (Laizhou Huayin Testing Instrument Co., Ltd., Laizhou, China, HVS-1000AB). During the measurement, the load was 10 gf, and the holding time was 15 s. Eight points were randomly selected on each specimen for measurement, and the hardness values were recorded. After removing the maximum and minimum hardness values of each test point, the average value was calculated as the hardness value of this group of specimens. The friction and wear tests were carried out on the experimental specimens using a friction and wear tester. The counterbody was a G5-grade ceramic ball with a diameter of 6.35 mm. The stroke length was 10 mm, the sliding speed was 10 mm/s, the applied normal load was 15 N, and the test duration was 1800 s, corresponding to 900 cycles and a cumulative sliding distance of 18 m. The tests were conducted under ambient atmospheric conditions at room temperature and pressure, with no significant vibration, no strong electromagnetic interference, no corrosive gases, and no dust interference. During the test, the direction perpendicular to the laser transverse scanning direction was selected as the reciprocating direction of the ceramic ball. The wear volume was calculated from the measured wear scar width, wear scar length, and the radius of the ceramic ball, assuming a flat wear scar geometry. The specific wear rate was calculated using the equation:

(2) $$K=\frac{V}{F\cdot s}$$

where *V* is the wear volume (mm^3^), *F* is the applied normal load (N), and *s* is the cumulative sliding distance (m); the corresponding unit of the specific wear rate is mm^3^/(N·m).

The electrochemical measurements were carried out by a CS310H electrochemical workstation (Wuhan Corrtest Instruments Co., Ltd., Wuhan, China) with a scanning rate of 0.5 mV/s at room temperature. A typical three-electrode system was used in electrochemical measurements: working electrode, platinum counter electrode, and reference electrode. The electrolyte was a 3.5% NaCl solution with a saturated calomel electrode. The open-circuit voltage of the sample was measured for 30 min to stabilize it. The judgment criterion for open-circuit potential is that its variation is within 2 mV within one minute. Electrochemical impedance spectroscopy measurements were carried out with an amplitude of 0.5 mV in the range of 10^5^–0.01 Hz. In order to ensure the accuracy of the results, the tests were repeated 3 times.

## 3. Results and Discussion

### 3.1. Surface Morphologies and Microstructure

**Figure 1:**
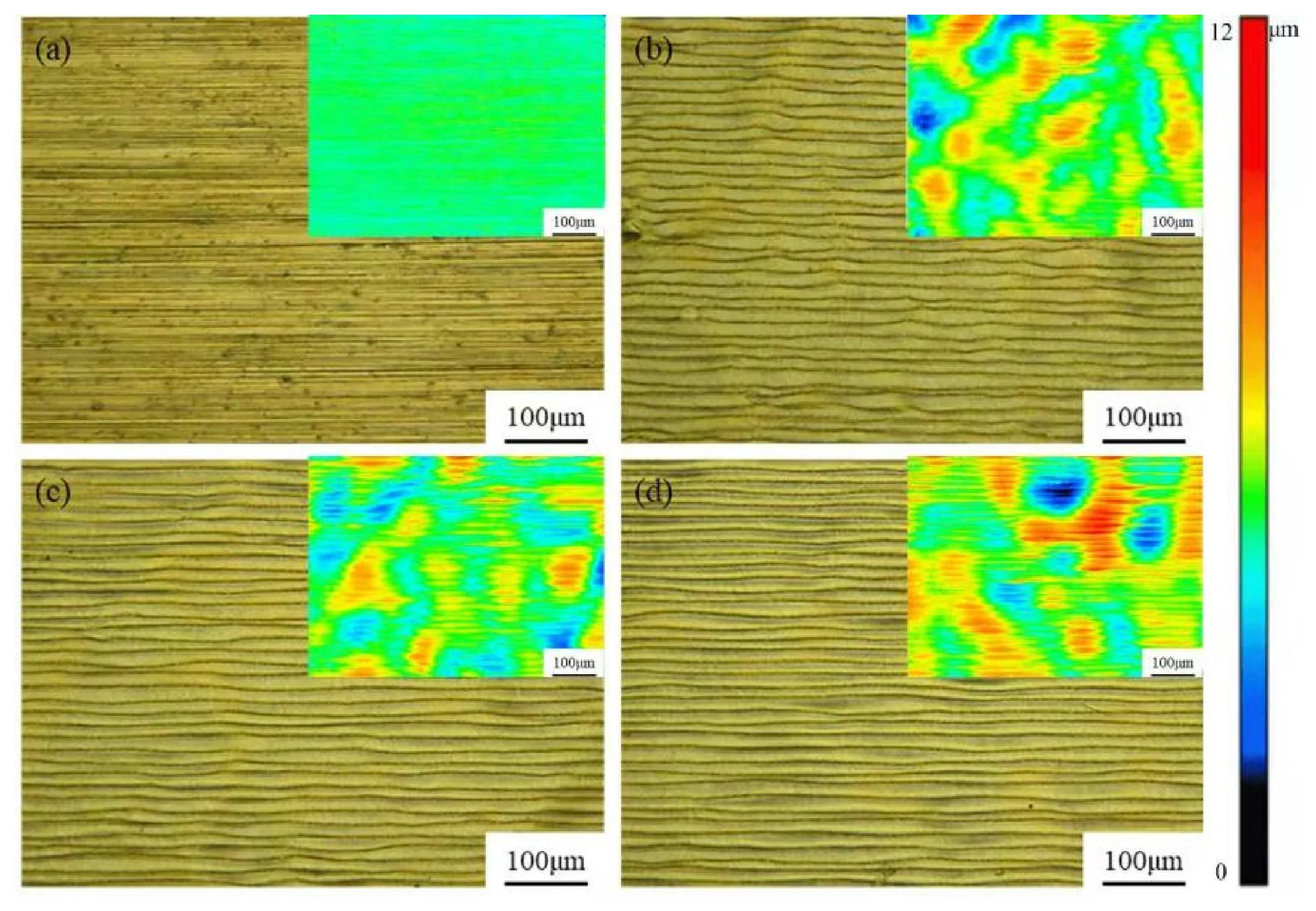
Surface morphology and three-dimensional surface morphology of aluminum bronze before and after LSM treatment. (**a**) L0; (**b**) L1; (**c**) L2; (**d**) L3.

Figure 1 shows the surface morphology of aluminum bronze observed by an optical microscope. As can be seen from Figure 1, the surface morphology of the aluminum bronze alloy changed obviously after LSM treatment. The surface scratches left by grinding in L0 (Figure 1a) completely disappeared after the melting and resolidification process, and a linear morphology with a fixed spacing was formed on the surface (Figure 1b–d). At the same time, the spacing between the lines of L1–L3 decreases, and the arrangement becomes denser as the laser power increases on the premise of a fixed line spacing (Figure 1b–d). As can be seen, the lines are actually formed by the connection of the edges of multiple laser molten pools, and the line spacing is the distance between the center points of the molten pools in adjacent horizontal paths. As the average laser power increases, the energy density of the laser increases, resulting in an increase in the range and depth of a single molten pool. Although the line spacing does not change, the distance between the edges of the molten pools in adjacent horizontal paths decreases, leading to a decrease in the spacing of the surface linear morphology of L1–L3 (Figure 1b–d). The altitude maps (inset in Figure 1a) show that the surface of L0 is relatively flat after grinding, with no obvious protrusions or depressions, and the overall height difference is small.

Figure 1 shows that the LSM-treated sample surfaces have areas of uneven height. To observe the distribution more intuitively, Confocal Laser Scanning Microscopy (CLSM, VK-X200K, KEYENCE Corporation, Osaka, Japan) was performed, with results shown in Figure 1. Blue areas represent depressions, and orange-red areas represent protrusions. The VK analysis software (Version 3.5.0.0) accompanying the CLSM was used to measure surface roughness, with results shown in Table 3. As seen in Figure 1, the L0 surface (Figure 1a) is relatively flat after grinding, with no obvious protrusions or depressions and a small overall height difference. Its measured surface roughness is 0.4 μm. After LSM treatment, L1–L3 (Figure 1b–d) exhibit noticeable uneven areas, leading to significantly increased surface roughness compared to L0. L3 has the highest surface roughness at 1.34 μm.

The reason is that aluminum bronze has a complex multi-phase microstructure with uneven phase distribution and different melting points. The LSM process is a complex high-temperature, transient, nonlinear process involving metal melting/solidification, evaporation, melt convection, heat conduction, etc., under the simultaneous action of various forces such as surface tension, recoil pressure, and gravity [18], resulting in uneven surface morphology.

**Figure 2:**
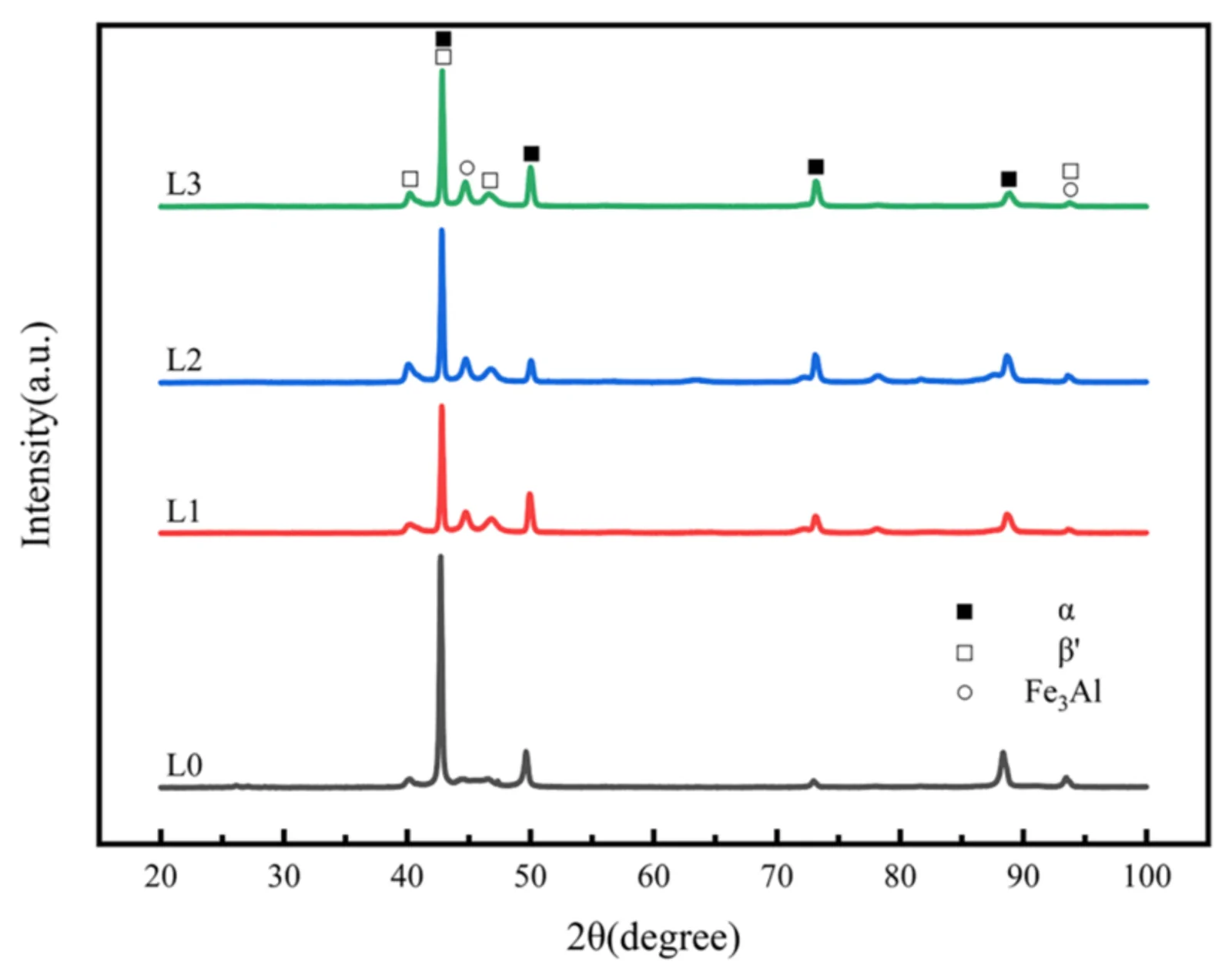
XRD patterns of aluminum bronze surface before and after LSM treatment.

The phase composition of as-cast aluminum bronze is complex. X-ray diffraction (XRD) was used for phase detection on the aluminum bronze surface before and after LSM treatment, with results shown in Figure 2. The phase identification of XRD patterns was based on the relevant literature [19,20] and performed using Jade (V6.2.9200(2)). The referenced PDF cards are as follows: #04-0836 (Cu), #28-0005\#49-1730 (Al-Cu), and #45-1203\#50-0955(Al-Fe). Figure 2 shows that the *β*′-phase-related peak intensities in the surface layer of L1–L3 first increased and then decreased after LSM treatment, which is qualitatively consistent with the microstructural observations described above. It should be noted, however, that quantitative phase fractions were not determined from the present XRD data, and the observed intensity trends are interpreted only as qualitative indications of relative phase changes. The reason is that the LSM process has an extremely short cooling/solidification time. The *β* phase, which would normally undergo eutectoid transformation to *α* + *κ* phases, is retained, forming a multi-phase remelted layer [21]. However, as laser power increases, the heat input to the aluminum bronze surface increases. Although the remelted layer thickness increases somewhat, the cooling/solidification time is prolonged, leading to the appearance of eutectoid α+κ phases, and their content continuously increases.

**Figure 3:**
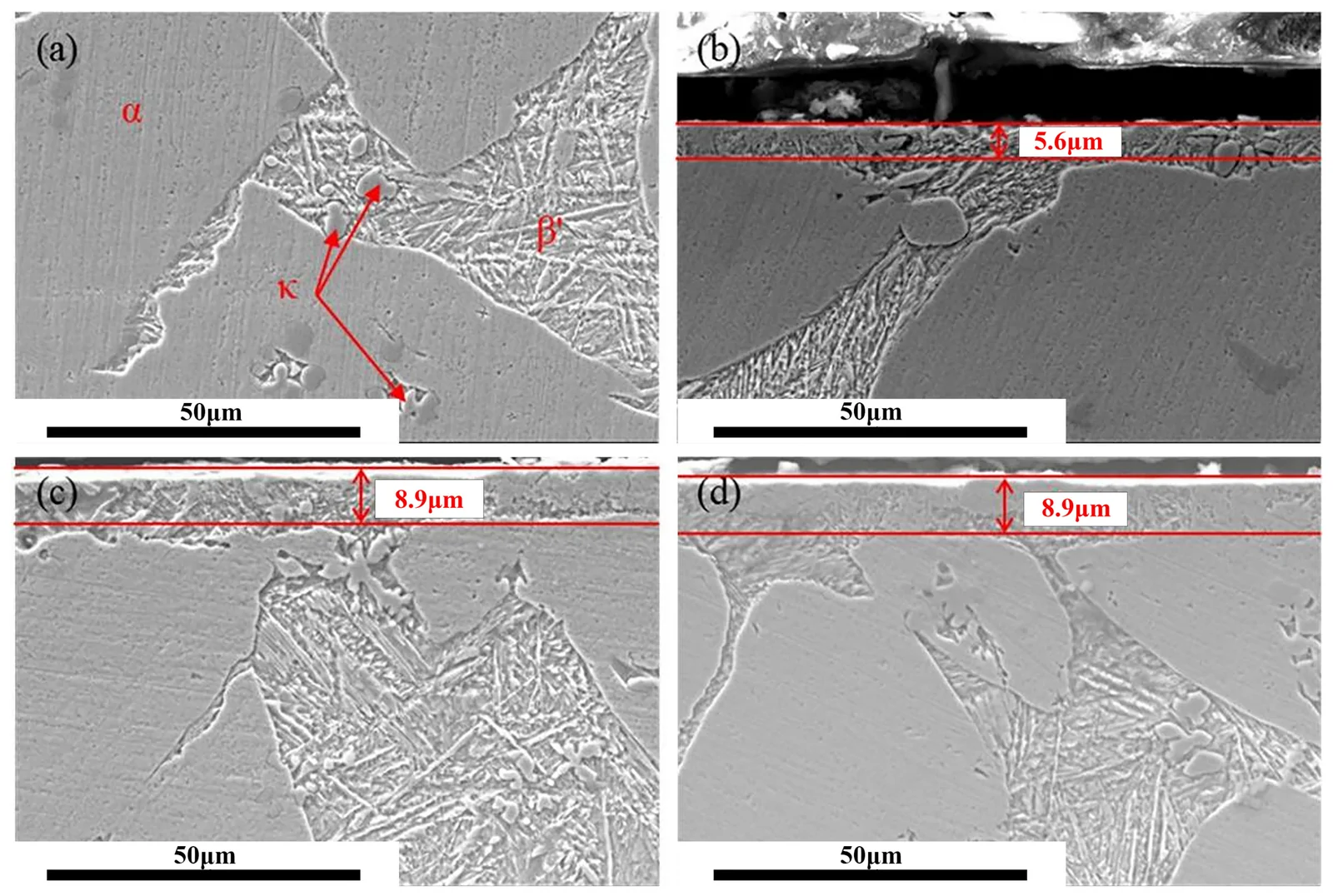
Cross-sectional microstructure and remelted layer thickness of aluminum bronze before and after LSM treatment. (**a**) L0; (**b**) L1; (**c**) L2; (**d**) L3.

Figure 3 shows the cross-sectional microstructure of aluminum bronze before and after LSM treatment. As shown in Figure 3a, it mainly consists of the matrix *α* phase, retained *β*’ phase, and *κ* phase. The *α* phase appears blocky, the *β*’ phase is typical lath martensite distributed in the matrix gaps, and the *κ* phase appears granular or petal-like, dispersed within the *α* or *β*’ phases. Figure 3 shows that after LSM treatment, remelted layers of varying thickness formed on the surfaces of L1–L3 (Figure 3b–d). Based on qualitative microstructural observations, the remelted layers primarily consist of the lath *β*′ phase and granular *κ* phase. The remelted layer thickness of L1 is about 5.6 μm, and the *β*′ phase appears noticeably finer than that in the substrate. The remelted layers in L2 and L3 are slightly thicker, approximately 8.9 μm, and the corresponding microstructural features appear coarser than those in L1. In addition to *β*′ and *κ* phases, the *α* phase is also observed in the remelted layers, especially in L3, where the *α* phase area appears significantly larger than the combined area of the *β*′ and *κ* phases based on the observed cross-section. However, these phase fraction estimates remain qualitative, as quantitative phase analysis was not performed.

**Figure 4:**
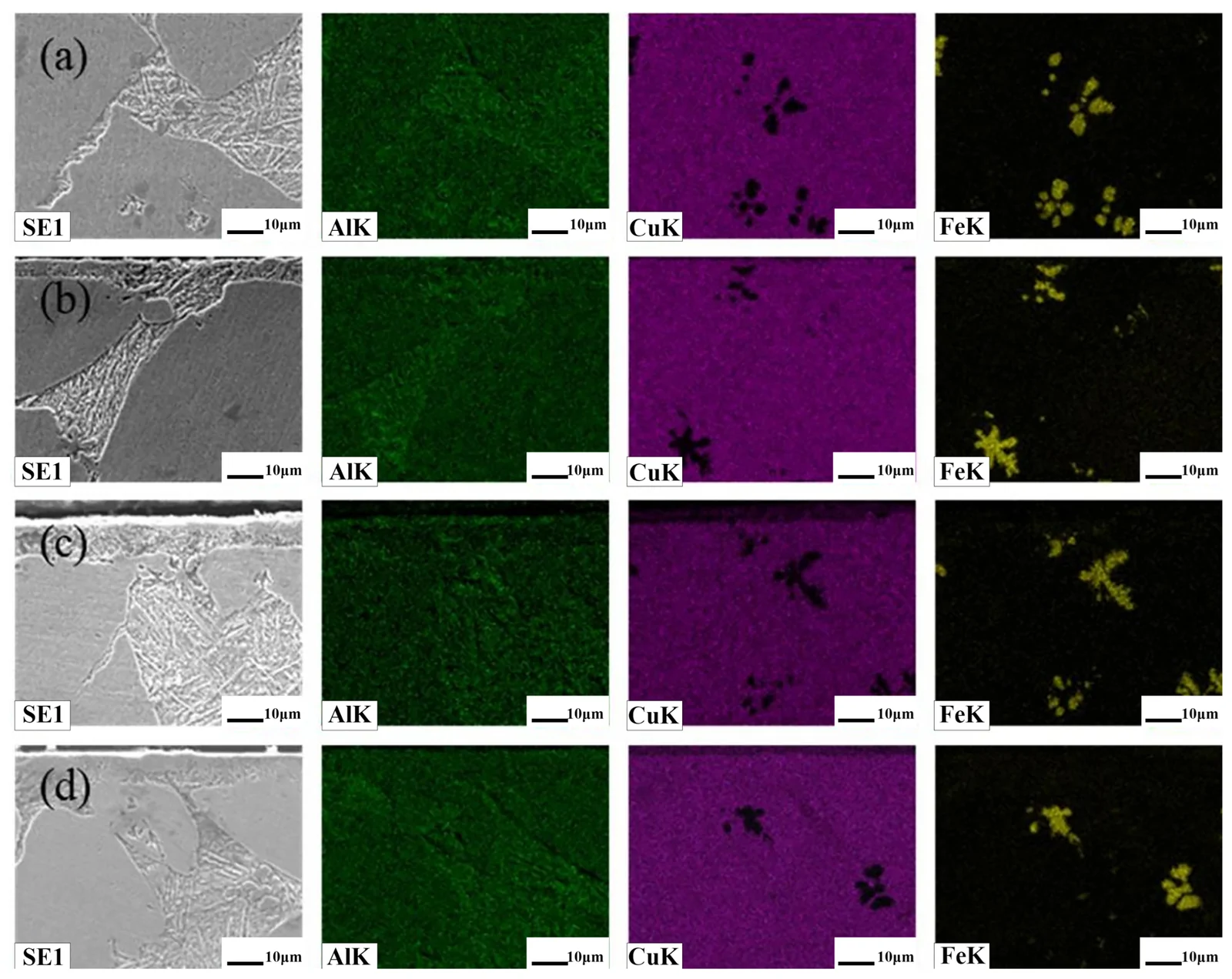
Cross-sectional elemental distribution of aluminum bronze before and after LSM treatment. (**a**) L0; (**b**) L1; (**c**) L2; (**d**) L3.

Elemental mapping was performed on the corresponding areas using EDS, mainly scanning for Cu, Al, and Fe, with results shown in Figure 4. In aluminum bronze, the *β*’ phase is mainly composed of Cu_3_Al, and the *κ* phase is Fe_3_Al. In elemental detection, the *β*’ phase shows Al enrichment, and the *κ* phase shows Fe enrichment. Figure 4 shows that the Al content in the L1–L3 surfaces increased relative to the matrix *α* phase. However, as laser power increased, the *β*’ phase content in the remelted layers of L1–L3 decreased, correspondingly leading to a decrease in Al content.

**Figure 5:**
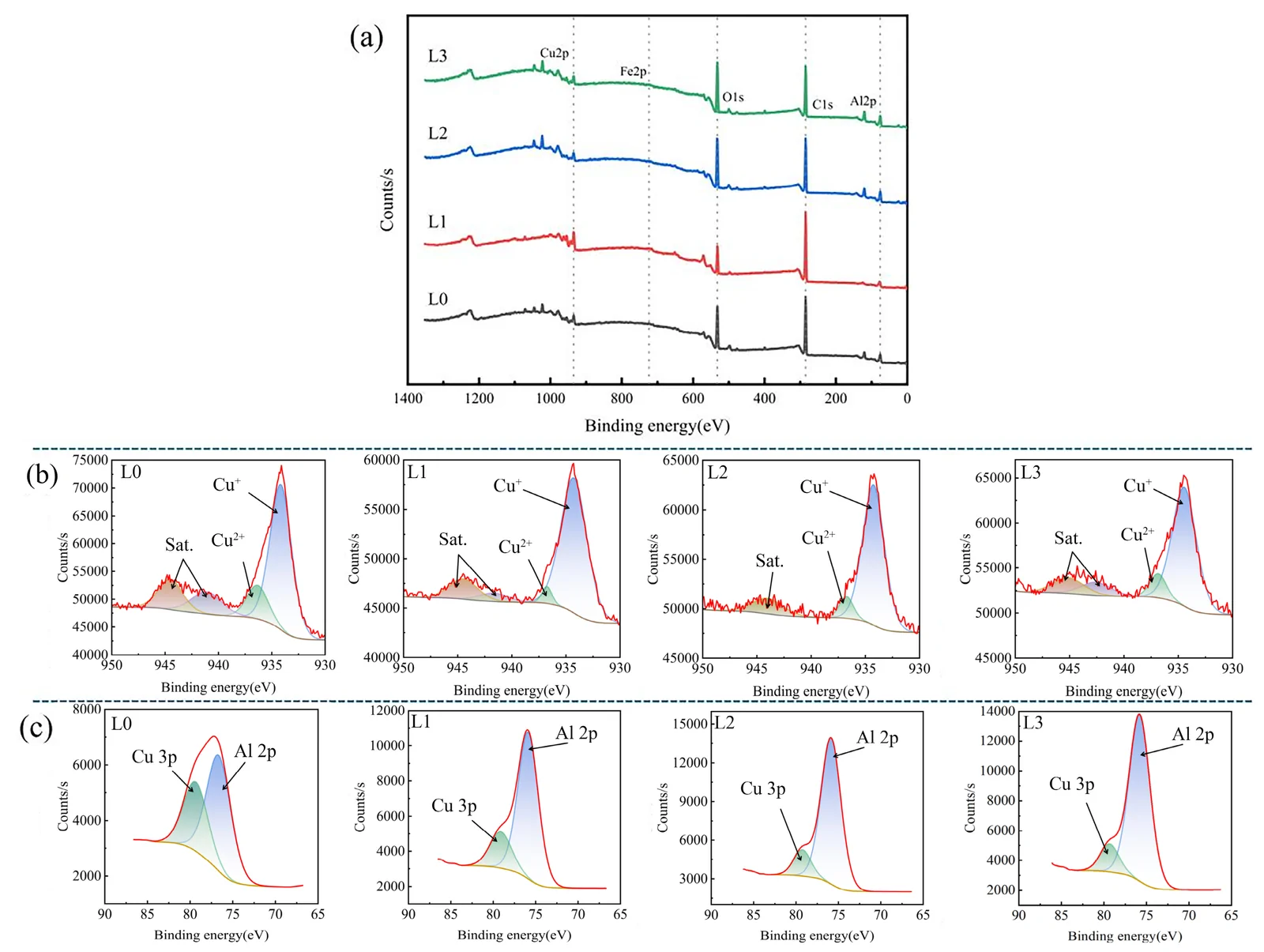
XPS spectra of aluminum bronze surface before and after LSM treatment. (**a**) XPS survey; (**b**) Cu 2p; (**c**) Al 2p.

X-ray photoelectron spectroscopy (XPS) was used to detect surface elements before and after LSM treatment, with results shown in Figure 5. The XPS survey spectra (Figure 5a) show that the main element types on the aluminum bronze surface did not change before and after LSM treatment, as LSM rapidly melts and solidifies to modify the surface without adding new material. However, variations in the intensities of the main photoelectron peaks were observed, which may suggest changes in the near-surface elemental composition. Since aluminum bronze is a multi-phase material, elemental distribution is closely related to phase distribution. These spectral differences are therefore tentatively interpreted as possible indications of surface phase transformation; however, changes in XPS peak intensity alone cannot be taken as direct quantitative evidence of bulk elemental concentration or phase fraction changes.

Figure 5b shows that the Cu 2p signal mainly consists of Cu^+^, Cu^2+^, and satellite peaks (characteristic of Cu^2+^, not observed in Cu and Cu^+^) [21,22,23,24]. The peak intensities for L0 are greater than those for L1–L3, and the satellite peak intensity decreased significantly after LSM treatment. These spectral changes may suggest a relative reduction in copper content within the XPS probing depth on the aluminum bronze surface after LSM, although they should not be interpreted as direct quantitative evidence of bulk composition changes. Figure 5c shows overlapping signals for Al 2p and Cu 3p. After peak fitting, a significant increase in Al^3+^ content on the aluminum bronze surface after LSM treatment is observed, indicating an increase in aluminum content. In summary, after LSM treatment, phase transformation occurred on the aluminum bronze surface, transforming phases with higher copper content into phases with higher aluminum content. However, given the limitations of XPS peak intensity analysis, the specific phase transformations require further confirmation by complementary phase-sensitive characterization.

### 3.2. Surface Hardness and Wear Resistance

**Figure 6:**
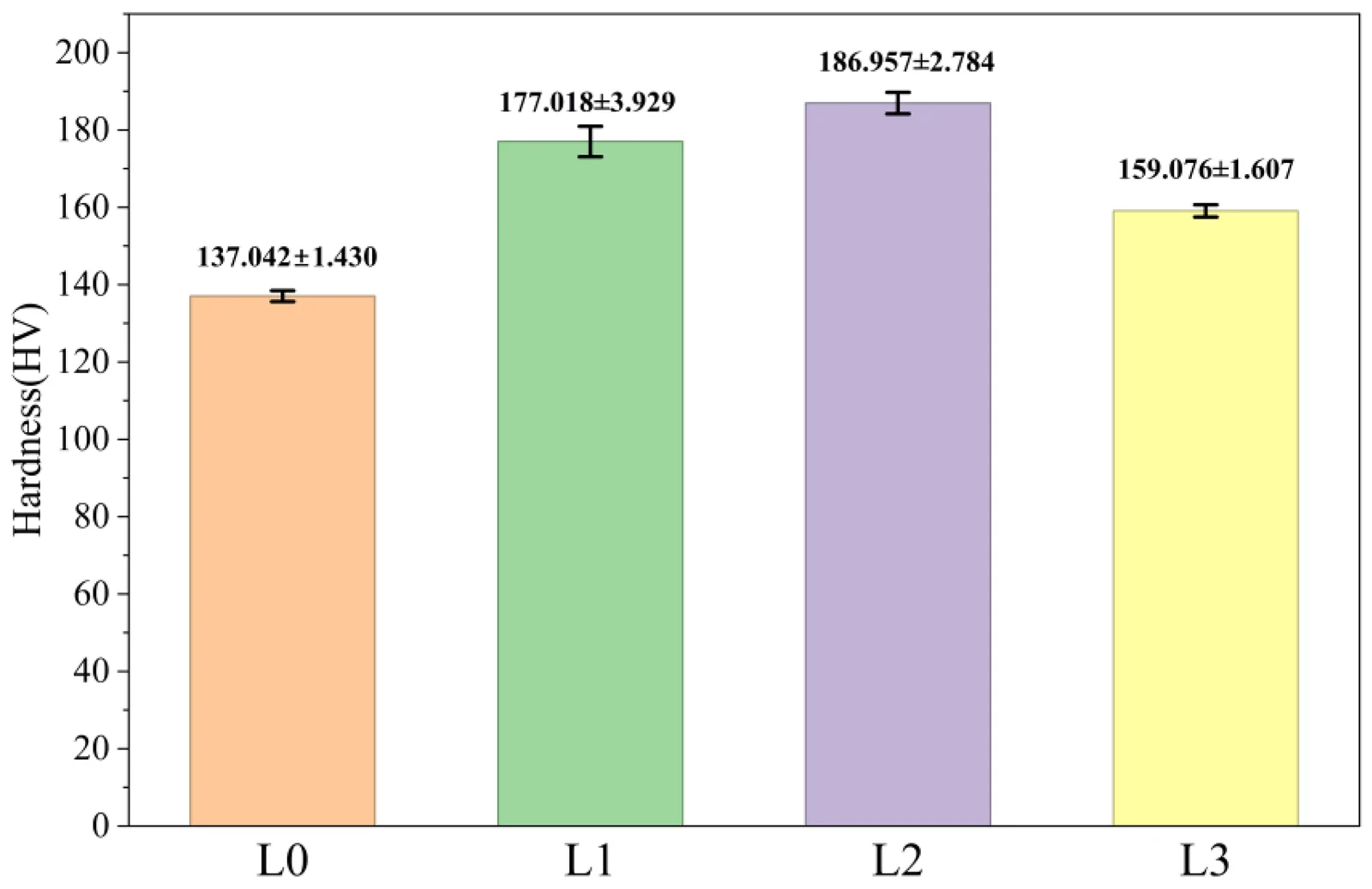
Hardness of aluminum bronze surface before and after LSM treatment.

Figure 6 shows the hardness of the aluminum bronze surface before and after LSM treatment. The surface hardness of L1–L3 significantly increased compared to L0. L2 had the highest hardness of 186.957 HV, a 49.915 increase over L0’s 137.042 HV. Based on the previous analysis of surface microstructure evolution, the *β*’ phase content on the aluminum bronze surface increased after LSM treatment. Compared to the more ductile α phase, *β*’ has higher hardness, leading to increased hardness in L1–L3.

**Figure 7:**
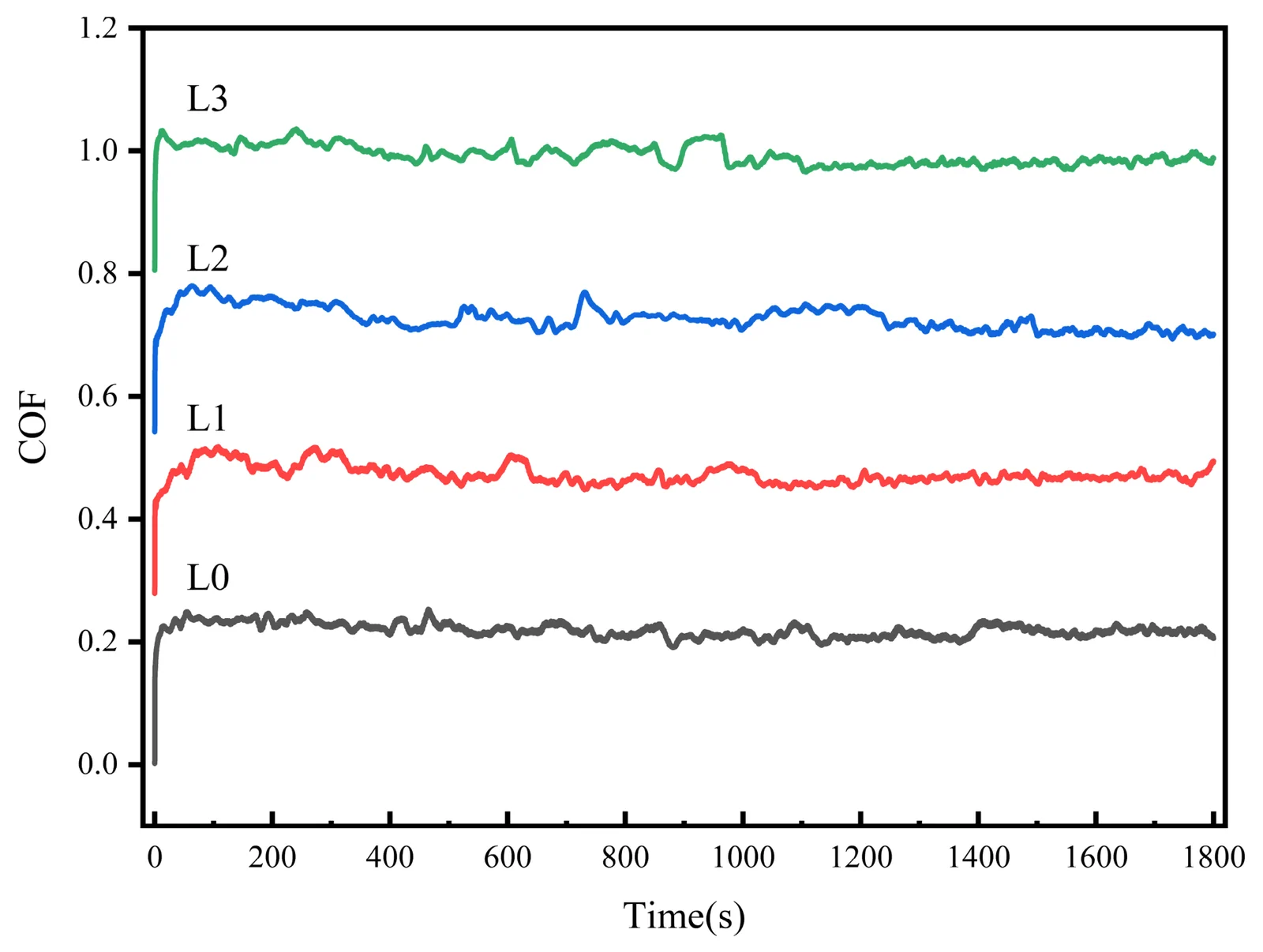
Friction and wear curves of aluminum bronze surface before and after LSM treatment.

Figure 7 shows the friction and wear curves for aluminum bronze before and after LSM treatment, which are relatively stable without abrupt changes. The curves in Figure 7 were plotted using Origin (Version 2026b) with a 10% automatic vertical stack function, in order to clearly present and compare each set of data within the same coordinate system. According to the average friction coefficient statistics, the average friction coefficients of the four samples are 0.219, 0.209, 0.196, and 0.202, respectively. The friction coefficients for L1–L3 are slightly lower than for L0, indicating improved wear resistance.

**Figure 8:**
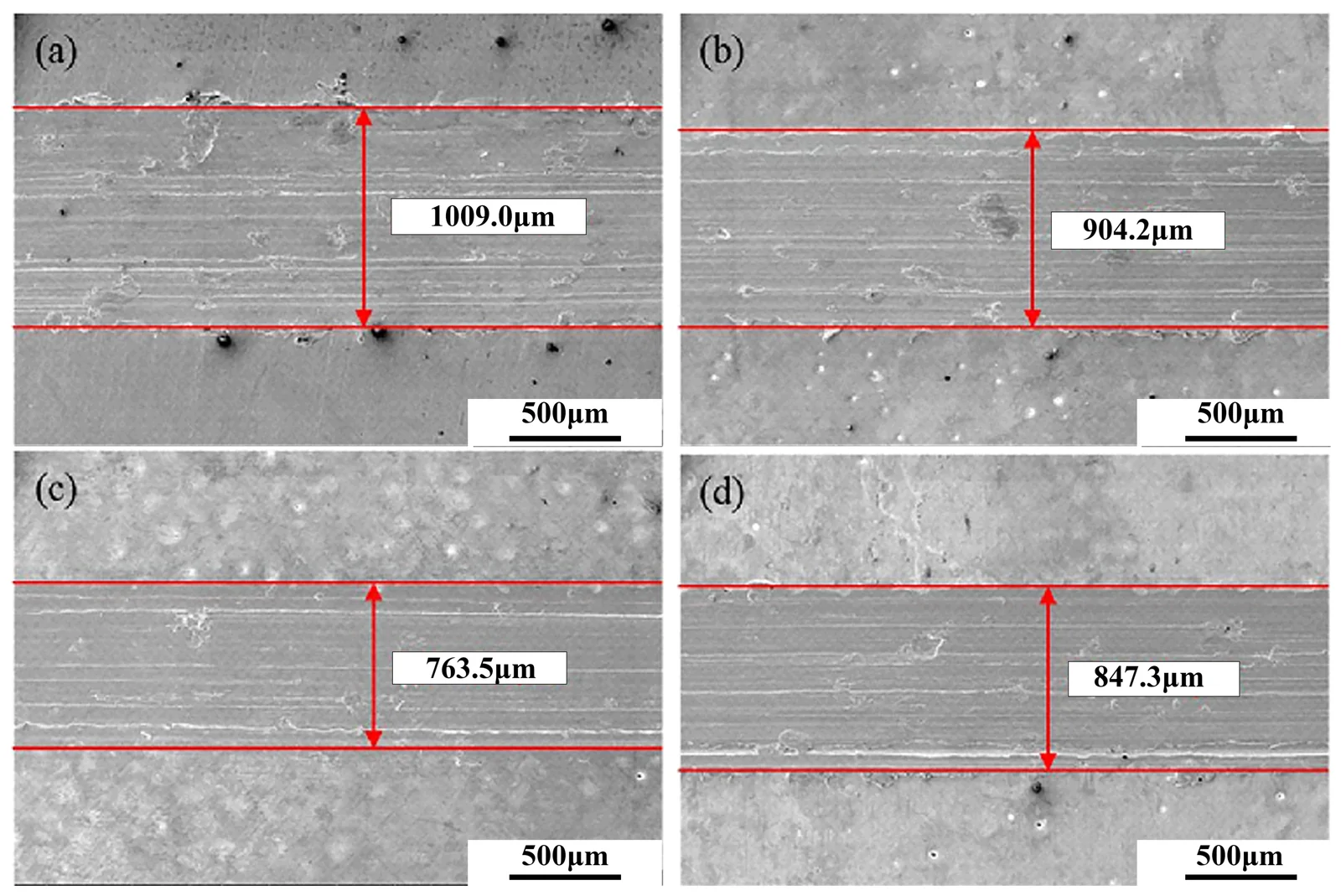
Wear scars on aluminum bronze surface before and after LSM treatment. (**a**) L0; (**b**) L1; (**c**) L2; (**d**) L3.

To more intuitively characterize the wear resistance, scanning electron microscopy (SEM) was used to observe the wear scars, as shown in Figure 8. The wear volume and specific wear rate are listed in Table 4. The wear scar width on L0 was the largest, about 1009.0 μm, and the scar contained many peeled-off areas, likely due to the detachment of the granular *κ* phase. The wear scar widths for L1–L3 were significantly smaller. Combined with the previous analysis, this is attributed to the increased *β*’ phase content after LSM treatment, leading to increased surface hardness and indirectly enhanced wear resistance. Further observation reveals that peeled-off pores inside the scars of L1–L3 were greatly reduced, and the internal structure was more uniform. L2 had the smallest wear scar width, about 763.5 μm, a 24.33% reduction compared to L0.

### 3.3. Electrochemical Corrosion Performance

**Figure 9:**
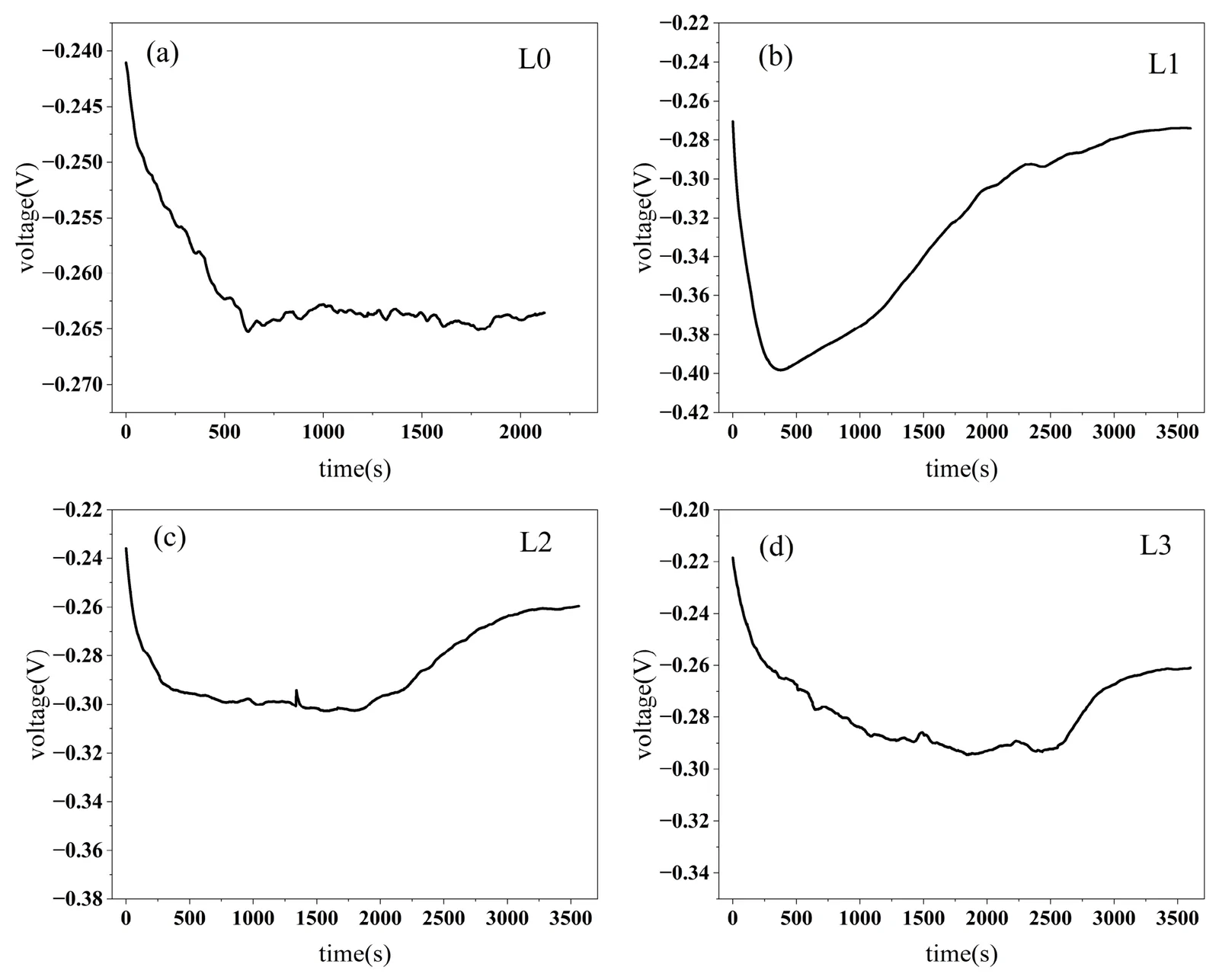
Open-circuit potential curve for samples. (**a**) L0; (**b**) L1; (**c**) L2; (**d**) L3.

**Figure 10:**
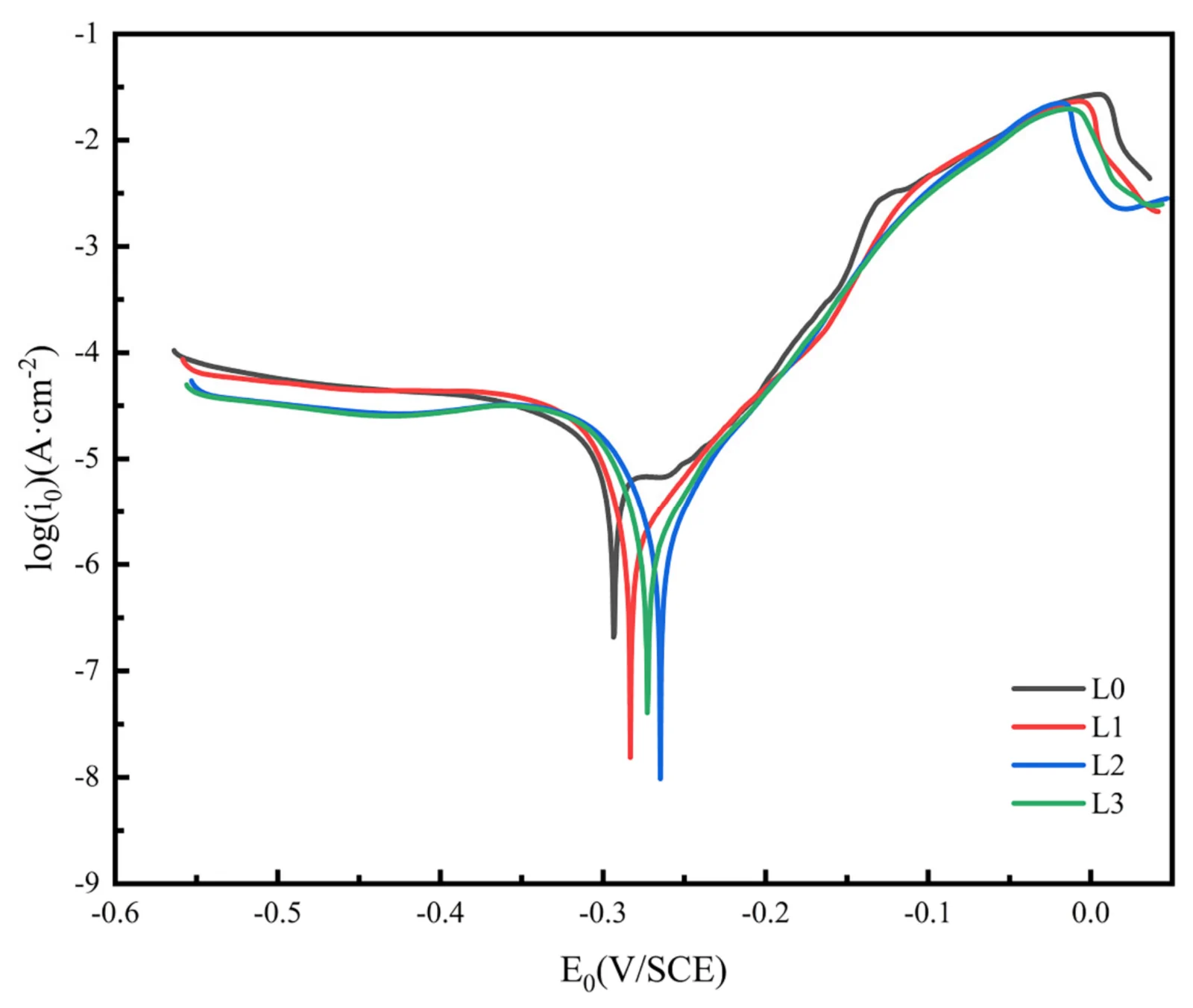
Polarization curves of aluminum bronze before and after LSM treatment.

Figure 9 shows the open-circuit potential curve for the samples. It can be seen that the open-circuit potential tends to be stable with time. Figure 10 shows a comparison of potentiodynamic polarization curves for aluminum bronze in a 3.5 wt.% NaCl solution before and after LSM treatment. In the cathodic region of the polarization curve, oxygen reduction primarily occurs. Over time, the corrosion current density continuously decreases, the corrosion potential gradually increases to the corrosion potential (*E*_corr_), and the oxygen reduction rate decreases. In the anodic region, oxidation corrosion reactions occur. The corrosion current density gradually increases with increasing corrosion potential until the material corrodes. When the corrosion potential reaches the passivation region, the corrosion current density stabilizes [25]. Figure 10 shows that the shapes of the potentiodynamic polarization curves for aluminum bronze before and after LSM are similar, consisting of cathodic oxygen reduction and anodic oxidation regions. After reaching a corrosion potential of about −0.05 V, they enter the passivation region. Compared to the untreated L0 specimen, the polarization curves of the LSM-treated specimens show a clear positive shift in corrosion potential (*E*_corr_), and they enter the stable passivation region at a lower corrosion potential, indicating higher thermodynamic stability and lower likelihood and rate of electrochemical corrosion.

Key electrochemical parameters from potentiodynamic polarization curves include corrosion potential (*E*_corr_), corrosion current density (*I*_corr_), and polarization resistance (*R*_p_). *E*_corr_ reflects the thermodynamic tendency for corrosion; a more positive *E*_corr_ indicates better corrosion resistance. *I*_corr_ reflects the kinetic reaction rate during corrosion; a lower *I*_corr_ indicates better corrosion resistance. *R*_p_ is related to the overall corrosion resistance; a higher *R*_p_ indicates better corrosion resistance [26]. To quantify these parameters, CS Studio5 software was used for fitting. The fitting results are summarized in Table 5.

From Table 5, the untreated L0 specimen has an *E*_corr_ of −0.29479 V, *I*_corr_ of 13.1445 μA/cm^2^, and *R*_p1_ of 1383 Ω·cm^2^. Specimens treated with three different LSM parameters (L1–L3) all have more positive *E*_corr_ and higher *R*_p_ values than L0, and lower Icorr values. L2 exhibits the best electrochemical parameters: *E*_corr_ = −0.26515 V, *I*_corr_ = 5.34145 μA/cm^2^, and *R*_p1_ = 3382.9 Ω·cm^2^.

**Figure 11:**
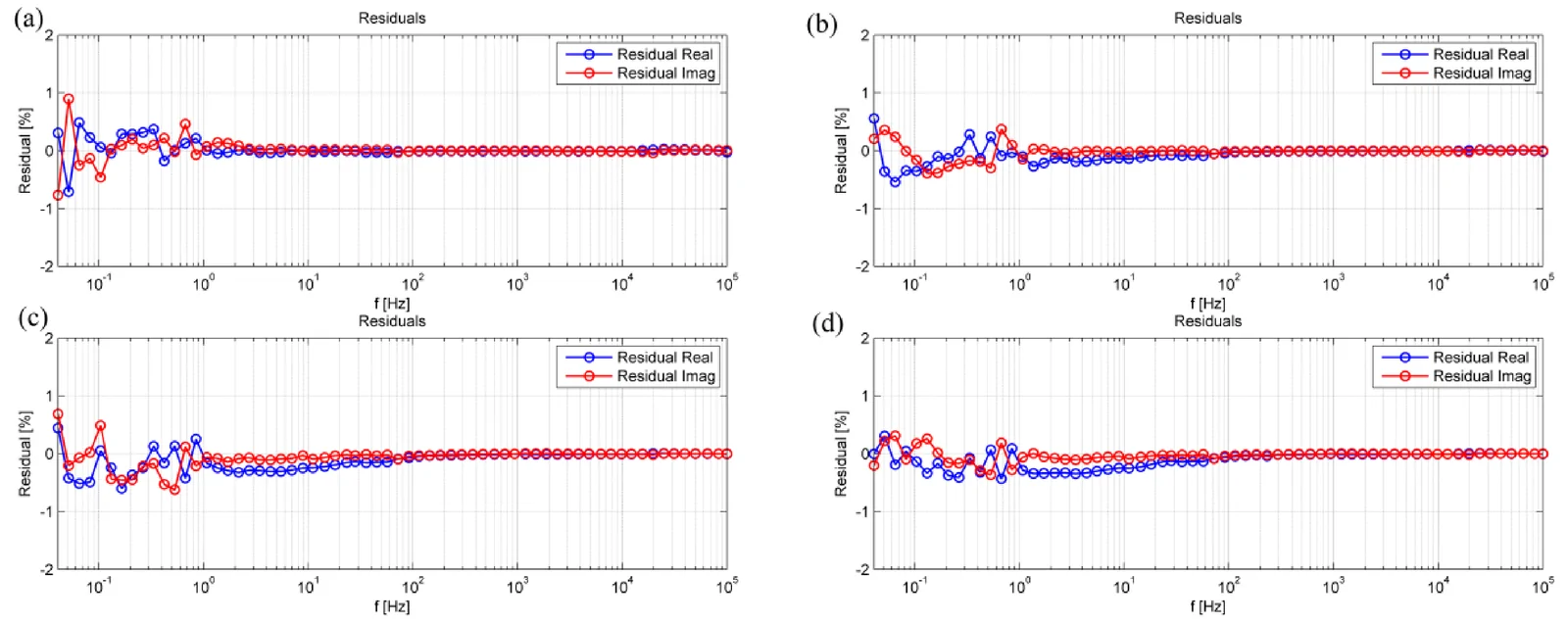
Kramers–Kronig (KK) verification residual curve: (**a**) L0; (**b**) L1; (**c**) L2; (**d**) L3.

**Figure 12:**
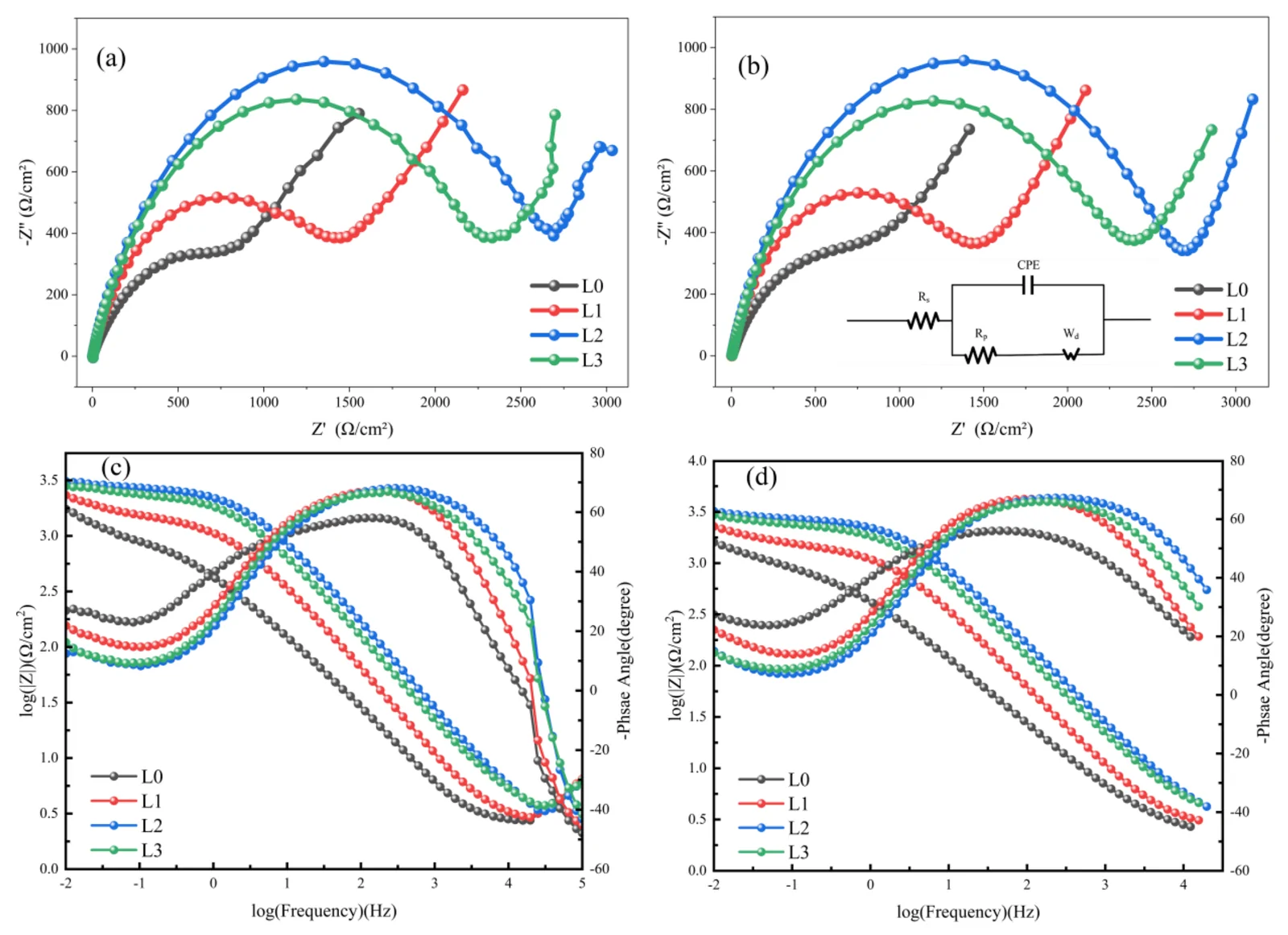
Impedance spectra and Bode plot of aluminum bronze before and after LSM treatment. (**a**) Nyquist plot; (**b**) fitted Nyquist plot; (**c**) Bode plot; (**d**) fitted Bode plot.

Kramers–Kronig (KK) verification was carried out to verify the validity of the EIS data. Figure 11 shows the residual curves of KK fitting, and all residual values are below 2%. Figure 12 shows the impedance spectra and Bode plot of aluminum bronze before and after LSM treatment. The shapes of the impedance spectra for different treatment parameters are similar, suggesting that LSM does not significantly alter the corrosion mechanism of aluminum bronze. The capacitive arc in the high-frequency region is related to the charge transfer resistance (*R*_ct_), film resistance (*R*_f_), and polarization resistance (*R*_p_) on the material surface. Generally, a larger radius of the capacitive arc indicates better corrosion resistance of the sample. The straight line in the low-frequency region corresponds to the Warburg impedance related to the diffusion process. The capacitive arc radii of L1–L3 samples after LSM treatment are all larger than that of the L0 sample, among which the L2 sample possesses the largest capacitive arc radius. The results reveal that LSM treatment effectively improves the corrosion resistance of NAB, and the L2 sample exhibits the optimal anti-corrosion performance, which is consistent with the conclusions obtained from potentiodynamic polarization tests.

The equivalent circuit [27,28] shown in Figure 12b was used with Zview (Version 3.3) to fit the EIS data. In the equivalent circuit, *W*_d_ represents Warburg impedance, and *R*_p_ denotes polarization resistance. To distinguish it from the polarization resistance in potentiodynamic polarization curves, the polarization resistance in electrochemical impedance spectra is denoted as *R*_p_′ hereinafter. CPE refers to the constant phase element, and its impedance is calculated as follows:

(3) $$Z_{\mathrm{CPE}}\;=\;{\left[Q{\left(j\omega \right)}^{n}\right]}^{-1}$$

where *Q* is a constant, *j* is the imaginary unit, and ω represents the angular frequency. *n* is a dimensionless constant. When *n* = 0, the CPE behaves with pure resistance; when *n* = 1, it acts as an ideal capacitor; when 0 < *n* < 1, it presents the characteristics of a non-ideal capacitor.

Table 6 gives the fitted circuit parameters from impedance spectra for aluminum bronze before and after LSM treatment. χ denotes the fitting coefficient, and its square value is adopted to evaluate the fitting quality. The Nyquist and Bode plots before and after fitting show good consistency, and all χ^2^ values are on the order of 10^−3^. This demonstrates that the selected equivalent circuit model achieves satisfactory fitting performance for the EIS data. As shown in Table 6, the *R*_p_′ value of sample L0 is 954.1 Ω·cm^2^. All LSM-treated samples L1–L3 exhibit higher *R*_p_′ values than sample L0. Among them, sample L2 has the maximum *R*_p_′ of 2692 Ω·cm^2^. This further confirms that LSM treatment can improve the electrochemical corrosion resistance of NAB, and sample L2 possesses the optimal electrochemical corrosion resistance.

**Figure 13:**
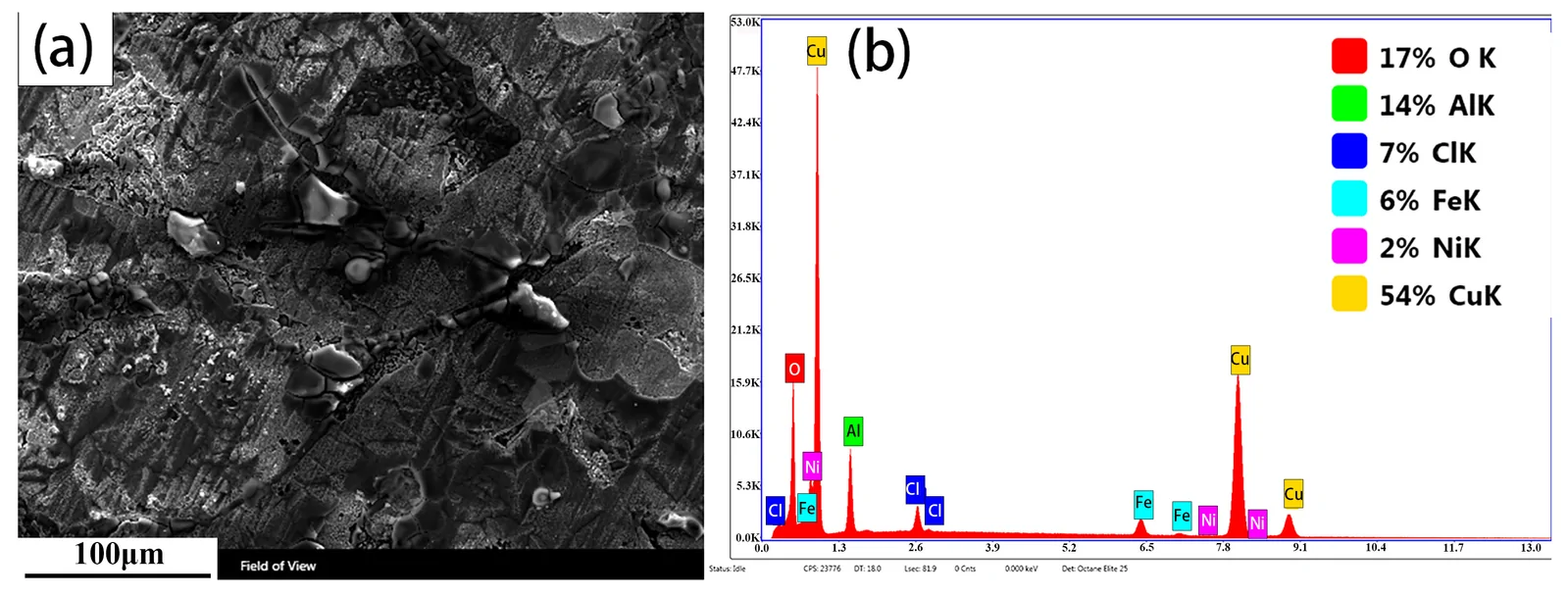
Surface morphology and corresponding EDS spectrum with elemental composition analysis of aluminum bronze before LSM treatment: (**a**) SEM surface morphology; (**b**) EDS spectrum and elemental composition analysis results.

**Figure 14:**
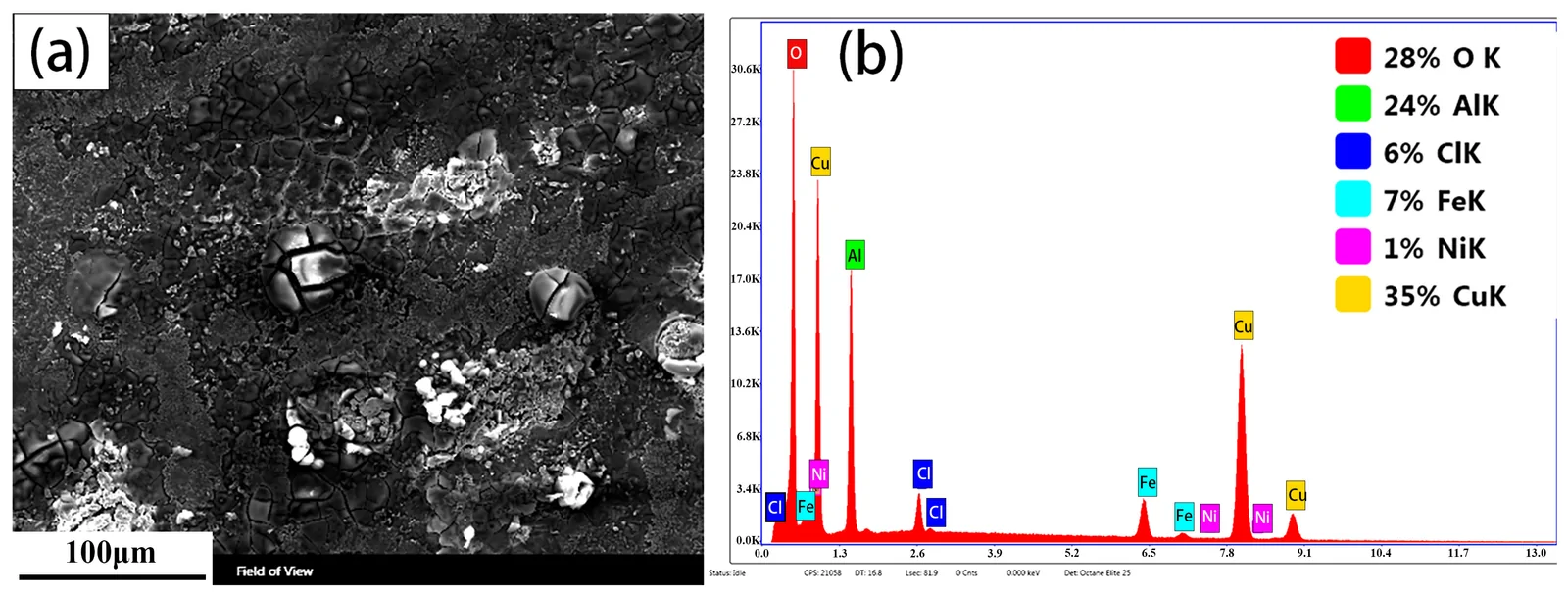
Surface morphology and corresponding EDS spectrum with elemental composition analysis of aluminum bronze after LSM treatment: (**a**) SEM surface morphology; (**b**) EDS spectrum and elemental composition analysis results.

Figure 13 and Figure 14 display the surface morphology, EDS mapping spectra, and elemental composition analysis of the electrochemical corrosion regions on the specimen surfaces before (L0) and after (L1) LSM treatment, respectively. As shown in Figure 13a, the specimen surface exhibits distinct cracks, and the corrosion products are loosely distributed in a flake-like manner. In contrast, Figure 14a reveals that the specimen surface is covered by a continuous and compact film-like corrosion product along with spherical nodular protrusions, where evident cracks are observable only at these spherical protrusions. A comparison between Figure 13b and Figure 14b indicates that the Al content after LSM treatment is 24%, significantly higher than the 14% recorded before LSM treatment. This suggests that the content of Al in the surface corrosion products is higher after LSM treatment, which may facilitate the formation of a dense Al-rich oxide film (presumed to be Al_2_O_3_) on the specimen surface, although phase-sensitive characterization would be required for conclusive identification.

**Figure 15:**
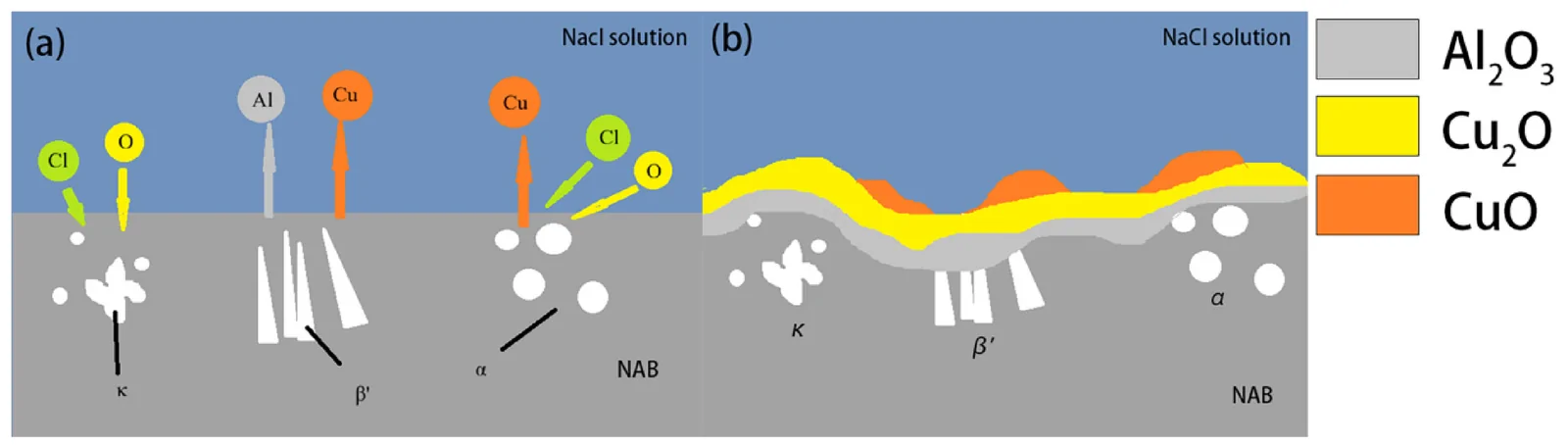
Schematic illustration of the electrochemical corrosion mechanism of aluminum bronze: (**a**) corrosion process diagram; (**b**) corrosion product diagram.

During the electrochemical corrosion of the aluminum bronze alloy in NaCl solution, the cathodic reaction is presumed to be oxygen reduction, while metal dissolution occurs at the anode. As illustrated schematically in Figure 15a, the α phase in the aluminum bronze matrix appears to be the first to undergo corrosion. The β’ phase subsequently releases Cu and Al ions outward, followed by the precipitation of the Al-rich κ phase. This process is suggested to result in the formation of a protective film rich in Al and Cu on the surface, with a relatively compact inner layer and a sparse outer layer (Figure 15b). The corrosion products rich in Al^−^ and Cu^−^ are presumed to include oxides such as Al_2_O_3_, Cu_2_O, or CuO [29,30,31,32,33], although this identification remains a hypothesis pending confirmation by phase-sensitive techniques.

The proposed cathodic reaction is as follows:

O_2_ + 2H_2_O + 4e^−^ → 4OH^−^ (4)

The proposed anodic reactions are as follows:

Cu − e^−^ → Cu^−^ (5)

Cu − 2e^−^ → Cu^2−^ (6)

Al − 3e^−^ → Al^3+^ (7)

The primary corrosion products of Cu are proposed to include Cu_2_O, CuO, and Cu_2_(OH)_3_Cl formed via further corrosion. The corrosion process is suggested to proceed as follows:

Cu + Cl^−^ → CuCl (8)

CuCl + Cl^−^ → CuCl^2−^ (9)

2Cu^−^ + 2OH^−^ → Cu_2_O + H_2_O (10)

2Cu_2_O + O_2_ + 2Cl^−^ + 4H_2_O → 2Cu_2_(OH)_3_Cl + 2H^+^ + 4e^−^ (11)

The primary corrosion product of Al is proposed to be Al_2_O_3_, and the main corrosion reactions are suggested as follows:

Al + 4Cl^−^ → AlCl^4−^ + 3e^−^ (12)

AlCl^4−^ + 3H_2_O → Al(OH)_3_ + 3H^+^ + 4Cl^−^ (13)

2Al(OH)_3_ → Al_2_O_3_ + 3H_2_O (14)

The Cu oxides are relatively sparse, whereas the Al oxide is considerably denser. This implies that a higher Al content on the surface is likely to promote the development of a dense oxide film, which is expected to hinder further corrosion effectively. However, the specific phase composition of the oxide film and the proposed reaction pathways require further experimental verification.

## 4. Conclusions

(1) Pulsed laser LSM treatment promotes phase transformation on the aluminum bronze surface. After LSM treatment, remelted layers of varying thickness form on the surface. Compared to the substrate, the remelted layers have reduced α phase content and increased *β*′ phase content. Among the samples, L2 exhibits the highest *β*′ phase content.
(2) Pulsed laser LSM treatment improves surface hardness and wear resistance of aluminum bronze. The phase transformation induced by LSM treatment forms a martensitic microstructure, enhancing the surface hardness and wear resistance. L2 has the highest hardness of 186.957 HV, a 36.42% increase compared to L0’s 137.042 HV. Its wear scar width is only 763.5 μm, a 24.33% reduction compared to L0.
(3) Pulsed laser LSM treatment improves the corrosion resistance of aluminum bronze. After LSM treatment, a martensitic microstructure forms on the aluminum bronze surface. This microstructural change is correlated with reduced susceptibility to selective phase corrosion and increased corrosion impedance; however, the specific contribution of β′ martensite to the improved corrosion resistance is proposed as a tentative interpretation, given the complex microgalvanic interactions among the *α*, *β*′, and *κ* phases. L2 shows the best corrosion performance with *E*_corr_ of −0.26515 V (increased by 0.03 V), *I*_corr_ of 5.34145 μA/cm^2^, and *R*_p1_ of 3382.9 Ω·cm^2^. Its impedance spectrum exhibits the largest capacitive loop radius, with *R*_p_′ of 2692 Ω·cm^2^, indicating superior corrosion resistance.

## Figures and Tables

**Table 1:** Main element content of aluminum bronze alloy (wt.%).

Element	Al	Fe	Ni	Mn	Cu
Measured composition	11.44	2.37	0.49	0.48	balance

**Table 2:** Method section of each sample.

Sample	L0	L1	L2	L3
Laser power (W)	0	120	140	160
Pulse energy (J)	0	1.20 × 10^−4^	1.40 × 10^−4^	1.60 × 10^−4^
Fluence (J/cm^2^)	0	2.39	2.79	3.18
Cumulative energy input (J)	0	1.596 × 10^−2^	1.862 × 10^−2^	2.128 × 10^−2^

**Table 3:** Surface roughness of aluminum bronze before and after LSM treatment.

Average Power (W)	L0	L1	L2	L3
Surface Roughness (μm)	0.4	1.1	1.08	1.34

**Table 4:** Specific wear rate of aluminum bronze surface before and after LSM treatment.

Sample	L0	L1	L2	L3
Wear scar width (μm)	1009.0	904.2	763.5	847.3
Wear volume (mm^2^)	0.274	0.200	0.115	0.158
Specific wear rate (mm^4^/N)	1.01 × 10^−5^	0.74 × 10^−5^	0.42 × 10^−5^	0.58 × 10^−5^

**Table 5:** Electrochemical parameters from polarization curves for aluminum bronze before and after LSM treatment.

	L0	L1	L2	L3
*E*_corr_ (V)	−0.29479	−0.27914	−0.26515	−0.26775
*I*_corr_ (μA/cm^2^)	13.1445	6.94985	5.34145	5.72255
*R*_p1_ (Ω·cm^2^)	1383	2590.35	3382.9	3158.55

**Table 6:** Fitted circuit parameters from impedance spectra for aluminum bronze before and after LSM treatment.

	L0	L1	L2	L3
*R*_s_/(Ω·cm^2^)	2.015	2.57	2.552	3.132
*Q*/(μF·cm^−2^·sn^−1^)	0.00049302	9.6696 × 10^−5^	3.8454 × 10^−5^	5.3982 × 10^−5^
*n*	0.67187	0.79425	0.78427	0.77643
*R*_p_′/(Ω·cm^2^)	954.1	1402	2692	2329
χ^2^/10^−3^	1.0175	2.8047	1.4123	2.8166

## Data Availability

The original contributions presented in this study are included in the article. Further inquiries can be directed to the corresponding author.

## Abbreviations

The following abbreviations are used in this manuscript:

LSM	Laser surface melting
